# The Diverse Roles of Reactive Astrocytes in the Pathogenesis of Amyotrophic Lateral Sclerosis

**DOI:** 10.3390/brainsci14020158

**Published:** 2024-02-04

**Authors:** Kangqin Yang, Yang Liu, Min Zhang

**Affiliations:** 1 Department of Neurology and Psychiatry, Tongji Hospital, Tongji Medical College, Huazhong University of Science and Technology, Wuhan 430030, China; kangkangy1999@163.com (K.Y.); liu_yang2014@hust.edu.cn (Y.L.); 2 Hubei Key Laboratory of Neural Injury and Functional Reconstruction, Huazhong University of Science and Technology, Wuhan 430030, China

**Keywords:** reactive astrocytes, amyotrophic lateral sclerosis, pathogenesis, astrocyte-targeting strategies

## Abstract

Astrocytes displaying reactive phenotypes are characterized by their ability to remodel morphologically, molecularly, and functionally in response to pathological stimuli. This process results in the loss of their typical astrocyte functions and the acquisition of neurotoxic or neuroprotective roles. A growing body of research indicates that these reactive astrocytes play a pivotal role in the pathogenesis of amyotrophic lateral sclerosis (ALS), involving calcium homeostasis imbalance, mitochondrial dysfunction, abnormal lipid and lactate metabolism, glutamate excitotoxicity, etc. This review summarizes the characteristics of reactive astrocytes, their role in the pathogenesis of ALS, and recent advancements in astrocyte-targeting strategies.

## 1. Introduction

Astrocytes, the most numerous and giant glial cells in the central nervous system (CNS), possess the unique ability to divide and proliferate throughout life. The cytosol of astrocytes exhibits a distinctive star-shaped morphology, housing a critical structural component known as the glial filament. Comprised of glial fibrillary acidic protein (GFAP), this intermediate filament is essential to the cytoskeleton and serves as a standard marker for astrocytes. Importantly, it is not entirely exclusive to astrocytes but also labels neural stem cells [1,2,3]. Apart from organizing the blood–brain barrier (BBB) and supporting, sequestering, and isolating neurons [1], these cells perform a multitude of vital biological functions. These include the metabolism, synthesis, and secretion of neurotrophic factors, regulation of neurotransmitters and calcium homeostasis, maintenance of mitochondrial function, participation in nervous system and circuit development, and regulation of the immune status of the CNS [1,2,4,5,6,7]. However, these functions are partially or wholly lost in reactive astrocytes.

Amyotrophic lateral sclerosis (ALS) is a fatal illness characterized by the degeneration of upper and lower motor neurons (MNs), with an average survival of 3–5 years [8,9]. Current treatments, such as riluzole, edaravone, AMX0035, and tofersen, can only temporarily extend survival [10,11,12,13,14,15]. The exact etiology and pathogenesis of the disease remain unknown, with proposed causes including neuroinflammation, oxidative stress, mitochondrial dysfunction, glutamate excitotoxicity, calcium homeostasis imbalance, metabolic abnormality, etc. [8,16,17]. The involvement of non-cell-autonomous processes, particularly reactive astrocytes, in the pathogenesis of ALS has been recognized [18,19]. In this review, we aim to summarize the contribution of reactive astrocytes in the pathogenesis of ALS and identify potential therapeutic targets.

## 2. Astrocytes in Pathological Conditions

### 2.1. Definition of Reactive Astrocytes

In the past, the response of astrocytes to abnormal events such as trauma, ischemia, infection, and tumor, epilepsy, and neurodegenerative and demyelinating diseases has been described using various terms, including astrocytosis, astrogliosis, reactive gliosis, astrocyte activation, astrocyte reactivity, astrocyte re-activation, and astrocyte reaction [20]. These terms can be replaced by the standard term “reactive astrocytes”, proposed to define the response process to pathological conditions, that is, under pathological conditions of the CNS, such as infection, trauma, or neurodegenerative disease, astrocytes undergo morphological, biochemical, transcriptional regulatory, molecular, and functional remodeling, ultimately losing most of their normal astrocytic functions and acquiring new neurotoxic or neuroprotective functions [20,21,22,23]. Compared to normal astrocytes, reactive astrocytes exhibit distinct morphological changes, including hypertrophy, elongation, process extension towards the injury site, and overlap of some three-dimensional structural domains [20,24]. It should be noted that the plasticity of healthy astrocytes, which are constantly activated by physiological signals from the CNS, should not be confused with changes in astrocyte responsiveness to pathological stimuli [20]. In this paper, the term “reactive astrocytes” and the above definition of reactive astrocytes will be used (Table 1).

### 2.2. Subsets/Heterogeneity of Reactive Astrocytes

In earlier studies, reactive astrocytes have been divided into neurotoxic and neuroprotective phenotypes, also known as A1 and A2 cell subpopulations [22] (Table 2). However, caution has been advised against using the oversimplified terms “neurotoxic” or “neuroprotective” when characterizing astrocyte phenotypes [20]. This is due to the limitations of these binary classifications in capturing the heterogeneity and diverse functions of reactive astrocytes, especially with advancements in technologies like transcriptomics and high-throughput sequencing [20,21,25]. Transcriptomic studies have revealed that reactive astrocyte phenotypes exhibit significant variability across different regions of the CNS and in response to various pathological stimuli [20,25]. In addition, a recent review [26] introduced an alternative classification scheme for astrocyte reactivity phenotypes. It categorizes them broadly into non-proliferative astrogliosis and proliferative astrogliosis. The former subtype typically occurs in neural tissue responding to pathology while maintaining its fundamental tissue architecture without overt damage [26]. This can be observed in tissue regions distant from focal lesions resulting from stroke, trauma, autoimmune attack, neurodegenerative changes, or diffuse neuroinflammation induced by peripheral exposure to microbial antigens such as lipopolysaccharide (LPS) [27,28,29,30]. On the other hand, the latter subtype exhibits anisomorphic features characterized by loss of domain, significant structural reorganization, and the potential diffuse alterations or development of new compact “limitans” borders surrounding evident fibrotic tissue lesions [31]. These changes can occur due to stroke, extensive trauma, infections, foreign bodies (including medical implants), autoimmune inflammation, neoplasms, or profound neurodegenerative processes [31,32,33]. This classification also possesses certain limitations similar to the previous categorization [26]. Therefore, a broader range of molecules is required to characterize these cells accurately [20]. When identifying astrocyte subpopulations, consideration must be given to multidimensional factors such as location, morphology, gene expression levels, specific cellular functions, and their demonstrated impact on pathological hallmarks to fully appreciate their heterogeneity [34].

### 2.3. Marker of Reactive Astrocytes

With the progression of sequencing technology, numerous astrocyte markers have been identified. However, the specific functions of these markers and their practical implications still need to be clarified, necessitating further exploration through in vivo and in vitro experiments. Several established markers commonly used to label reactive astrocytes and emerging markers with slightly defined functions are described below.

#### 2.3.1. GFAP

As previously delineated, GFAP is an essential protein component of astrocyte intermediate filaments, contributing to the cytoskeletal organization, and serves as the most extensively employed marker of reactive astrocytes [2,7,20]. A prevalent attribute of numerous reactive astrocytes, albeit not all, present in various CNS disorders is the elevation of GFAP due to the upregulation of GFAP mRNA and protein rather than local recruitment or proliferation of astrocytes [34,35]. GFAP is also a sensitive indicator of an early injury response, detectable even without apparent neuronal death [36]. Furthermore, the severity of the injury is correlated with the quantity of GFAP expression in reactive astrocytes [20,37]. Notably, elevated GFAP levels are necessary but insufficient for reactive astrocyte classification, suggesting that increased GFAP levels occur due to pathological stimuli and regional differences in astrocytes, initial GFAP levels, and physiological stimuli [20]. For instance, in a healthy mouse brain, astrocytes in the hippocampus exhibit higher levels of GFAP than those in the cortex, thalamus, or striatum. However, this does not imply that astrocytes in the hippocampus are inherently more reactive [38,39,40]. Additionally, GFAP expression is influenced by physiological stimuli such as physical activity, exposure to enriched environments, glucocorticoids, and fluctuations in the circadian rhythm in the suprachiasmatic nucleus of the optic cross [41,42]. Moreover, GFAP is not exclusively derived from astrocytes but can also be produced by progenitor cells, depending on the stage of development [3]. Hence, changes in GFAP levels reflect a response to pathological stimuli and adaptation to physiological stimuli and regional differences.

#### 2.3.2. Complement C3

In the central nervous system, complement component 3 (C3) is primarily synthesized by astrocytes [43] and has been used as a marker for type A1 or neurotoxic astrocytes [22]. In conjunction with GFAP, it labels reactive astrocytes and exerts neurotoxicity in neurodegenerative diseases, such as Alzheimer’s disease (AD), ALS, multiple sclerosis (MS), and Parkinson’s disease (PD), as well as in infectious diseases and spinal cord injury [44,45,46,47]. Under physiologic conditions, C3 secreted by astrocytes is involved in the complement cascade and mediates synaptic elimination during the development of the CNS [48]. However, when the CNS is subjected to infections, injuries, and neurodegenerative diseases, the secretion of C3 by reactive astrocytes significantly increases, activating the complement cascades abnormally to eliminate normal synapses and resulting in the loss of neurons and damage to cognitive function [47]. Additionally, C3 also enhances superoxide production and mediates oxidative stress injury in the nervous system [49]. The oxidative stress induced by LPS in chronic neuroinflammation is significantly reduced without C3 expression [49].

#### 2.3.3. Other Markers

TNF-related apoptosis-inducing ligand (TRAIL), also known as tumor necrosis factor superfamily member 10 (TNFSF10), is a member of the TNF superfamily, contributing to apoptosis through both extrinsic and intrinsic signaling pathways. Initially, it was believed to specifically target tumor cells and was absent in the healthy CNS. However, recent studies have demonstrated that astrocytes, microglia, and neurons can express TRAIL within the CNS under pathological conditions [50]. Notably, the function of TRAIL^+^ astrocytes is versatile. For instance, in experimental autoimmune encephalomyelitis (EAE), which is a mouse model used for studying MS, TRAIL^+^ astrocytes, acting as anti-inflammation astrocytes, induce CD4^+^ T cell apoptosis to alleviate neuroinflammation [51]. Conversely, TRAIL^+^ astrocytes exhibit toxicity towards neurons in the AD mouse model, directly inducing the apoptosis of neurons [52,53].

Reactive astrocytes also express several nonspecific markers [20], including those associated with the cytoskeleton (nestin, synemin, vimentin), cellular metabolism (ALDOC, BLBP/FABP7, MAO-B, TSPO), membrane channels (EAAT1 and 2, KIR4. 1), secreted proteins (CHI3L1/YKL40, Lcn2, Serpina3n/ACT, MT, THBS-1), signal transduction and transcription (NFAT, NTRK2/TrkB IL17R, S100B, SOX9, STAT3), molecular chaperones (CRYAB, HSPB1/HSP27), etc. It should be highlighted that the markers mentioned above are not exhaustive, and they have yet to be employed as a unique marker of reactive astrocytes due to their inability to distinguish between specific types of reactive astrocytes. Consequently, numerous additional markers need to be identified.

### 2.4. Functions of Reactive Astrocytes

Reactive astrocytes exhibit a significant loss of functional capabilities compared to their regular counterparts, characterized by reduced trophic support, neurotransmitter uptake, synapse formation, gap junction coupling, and altered neuronal activity [20,54]. These deficits are accompanied by increased immune cell infiltration and irregular calcium transients [54]. Reactive astrocytes can acquire novel neurotoxic or neuroprotective functions depending on the specific pathological condition [21,23,54]. For instance, in a transgenic mouse model targeting reactive astrocyte ablation, CNS tissue experiences a significantly more severe disruption, demyelination, neuronal and oligodendrocyte death, and pronounced motor deficits, along with an inability to repair the blood–brain barrier, in comparison to the non-transgenic mouse model after mild or moderate stab or crush spinal cord injury [55]. Notably, the functions of reactive astrocytes are not always constant. After cerebral ischemia, these cells exhibit a protective role in the early stage by secreting neurotrophic substances and antioxidants and forming glial scars to limit the spread of the immune response. However, in the later stages, the formation of a glial scar impedes neurological recovery [56]. In summary, when the CNS experiences secondary degeneration caused by trauma or ischemia, reactive astrocytes may provide protection in the early stage by repairing the blood–brain barrier, restricting neuroinflammation, and preserving motor functions. Nonetheless, astrocytic scar formation is detrimental to axonal regeneration in the later stages [57]. Conversely, in neurodegenerative diseases (NDs) such as AD, PD, MS, Huntington’s disease (HD), and ALS, abnormal protein accumulation, excessive production of inflammatory factors and reactive oxygen species (ROS), and disruption of ion homeostasis and metabolism in reactive astrocytes contribute to a persistent inflammatory environment and neuron death [34,54]. The extent of the protective influence of reactive astrocytes in NDs during various disease stages has yet to be thoroughly investigated.

### 2.5. Link between Reactive Astrocytes and Environmental Elements

The development of neuropathological conditions, such as ALS, is influenced by environmental factors, such as the presence of heavy metals and pesticides. In today’s rapidly industrializing and modernizing world, heavy metal pollution has emerged as a well-recognized public health concern that impacts daily life, including food, water sources, air quality, and occupational exposure [58,59]. Excessive intake of heavy metals can lead to neurotoxicity and subsequent neurological disorders [60,61,62]. As previously mentioned, astrocytes play a pivotal role in maintaining homeostasis in the central nervous system and safeguarding neurons against various types of harm caused by heavy metal accumulation [7,61]. However, this protective function also makes astrocytes susceptible to the neurotoxicity of heavy metals. This vulnerability manifests through distributions in blood–brain barrier integrity, elevated levels of ROS, pro-inflammatory factors, impairment of mitochondrial respiration, and abnormalities in glutamate and lipid metabolism. These effects have been demonstrated through numerous experiments conducted both in vivo and in vitro [60,61,62,63,64,65,66,67]. For instance, Shi Fan et al.’s study [63] involved exposing rats to drinking water containing lead acetate (PbAc) for nine continuous weeks, which impaired learning memory and exploratory abilities. Additionally, expression levels of GFAP, along with other genes associated with reactive astrocytes affected by neurotoxicity, were significantly elevated compared to the control group. Subsequent experiments involved administration of PbAc to MA-c cells, an astrocyte cell line, confirmed these findings while revealing that NF-κB transcription factor regulates astrocyte activation following lead exposure [63]. Despite substantial evidence from experimental studies supporting these conclusions, however, little information is available regarding the cumulative effects of heavy metals on human astrocytes [61]. Given the indispensable role of pesticides in agricultural development, humans are exposed to these chemicals through various means such as occupational activities, agricultural practices, domestic use, and air, water, soil, and food contamination. Similar to heavy metals, this exposure leads to disruption of the BBB and activation of astrocytes, based on numerous studies conducted in vivo and in vitro [68,69,70,71,72,73,74]. Furthermore, research has indicated that Parkinson’s disease protein 7 (PARK7/DJ-1), found in astrocytes, plays a crucial role in regulating the neurotoxic consequences caused by the pesticide rotenone [75,76,77]. Additionally, maintaining astrocyte homeostasis is closely associated with other environmental factors such as gut microbiota, composition intake of dietary components, and air pollution [78,79,80,81].

## 3. Reactive Astrocytes Are Toxic to MNs in ALS

Reactive astrocytes in ALS display morphological modifications, such as hypertrophy, process elongation, and partial overlap of specific features of these cells (protrusions, branches, or other morphological characteristics) in three-dimensional space [20]. Moreover, these cells undergo chemical remodeling characterized by increased transcription of pro-inflammatory factors and oxidation particles [82]. They also exhibit molecular remodeling, with markedly elevated expression of GFAP and C3 compared to naive astrocytes [83]. Numerous in vivo and in vitro studies have corroborated the significant elevation of C3 mRNA and protein levels in mouse models of ALS, as well as in patients with familial and sporadic ALS (SALS and FALS) [22,83,84,85]. Furthermore, inhibition of C3 release from reactive astrocytes has been shown to reduce neuronal damage [86]. One previous study has demonstrated that astrocytes derived from both sporadic and familial ALS patients exhibit an equivalent level of toxicity toward motor neurons [82]. In this investigation, SALS and FALS astrocytes were obtained from postmortem spinal cord neural progenitor cells (NPCs), which were then supplemented with 10% fetal bovine serum to induce differentiation into astrocytic. This innovative model system was employed to elucidate the underlying molecular mechanisms and evaluate potential therapeutic approaches for SALS [82]. The researchers co-cultured these differentiated astrocytes with mouse embryonic stem-cell-derived motor neurons, observing an accelerated demise of motor neurons in this co-culture setup while noting a significant upregulation of twenty-two inflammatory genes in both FALS and SALS astrocytes [82]. Additionally, when these were co-cultured with GABAergic neurons, they did not exert any influence on them. These findings suggest that astrocytes target and impair motor neurons [19,82,87].

Frontotemporal dementia (FTD) encompasses a spectrum of disorders characterized by the progressive degeneration of the frontal and temporal lobes in the brain, resulting in alterations in personality, behavior, and language. Coexistence or shared clinical, genetic (*SOD1*, *TARDBP*, and *C9ORF72* et al.), and pathological features (TDP-43 inclusions in astrocytes and neurons) have been observed between ALS and FTD [88]. Previous studies have demonstrated that astrocytes from postmortem patients with ALS/FTD also exert detrimental effects on motor neurons [89,90,91]. Astrocyte dysfunction specific to ALS/FTD is comparable to that seen in ALS without FTD; however, there are some distinctions [92]. For example, individuals with both ALS and FTD exhibit more pronounced increased blood–brain barrier permeability associated with poor prognosis compared to those solely affected by ALS [93]. In a clinical trial comparing GFAP levels within serum samples taken from participants experiencing cognitive and/or behavioral impairment or FTD versus those diagnosed only with ALS, the results showed significant variation [88]; however, no difference was noted among various types of clinical presentations for individuals diagnosed with only ALS regarding GFAP levels within their serum samples [88].

Recent investigations have indicated distinct roles for astrocytes derived from both the motor cortex (MC) and the spinal cord (SC) of newborn *SOD1^G93A^* mice during disease progression stages [94,95,96]. Spectrophotometric and cytofluorimetric analyses revealed elevated redox stress, reduced antioxidant capacity, and relative mitochondrial respiratory uncoupling in MC *SOD1^G93A^* astrocytes. In contrast, SC mutated cells exhibited enhanced resistance against oxidative damage, attributed to augmented antioxidant defense [94]. However, the most extensively studied astrocytes throughout this manuscript are predominantly derived from the spinal cord.

In general, the detrimental effects of reactive astrocytes are not only mediated by mitochondrial dysfunction, calcium homeostasis dysregulation, and endoplasmic reticulum stress but also exacerbated by metabolic dysfunction. This exacerbation triggers neuroinflammatory responses and releases various toxic factors, including polyphosphate, glutamate, lactate, and lipids, which directly act on motor neurons.

### 3.1. Mitochondrial Dysfunction

Prior research has observed a disruption of the mitochondrial respiratory chain in astrocytes derived from *SOD1^G93A^* rats, characterized by diminished oxygen utilization, absence of ADP-dependent respiratory control, and reduced membrane potential. This leads to elevated oxygen radicals and nitric oxide levels, contributing to motor neuron demise [97,98,99]. There is a notable reduction in motor neuron survival when employing mitochondrial respiration inhibitors in non-transgenic astrocyte cultures, particularly azide-dependent inhibition of cytochrome c oxidase and fluorocitrate-dependent inhibition of aconitase [97]. This finding suggests that the survival of motor neurons depends on the mitochondrial function in astrocytes. Furthermore, the neurotoxic phenotype can be mitigated by restoring mitochondrial respiration in astrocytes by using antioxidants Mito-Q and Mito-CP [97]. In other in vivo studies [100,101], it was found that the administration of dichloroacetate (DCA), a drug enhancing mitochondrial function by stimulating the activity of the pyruvate dehydrogenase (PDH) complex, improves survival and motor performance while reducing MN degeneration and gliosis in the *SOD1^G93A^* rat model. Furthermore, when DCA was used in astrocyte–motor neuron co-cultures (consisting of astrocytes derived from the spinal cord of an *SOD1^G93A^* rat and motor neurons derived from embryonic day 15 rats), phosphorylation of PDH decreased, leading to enhanced mitochondrial coupling and increased motor neuron survival [101].

### 3.2. Disturbance of Ca^2+^ Homeostasis

The resting state of astrocytes is contingent on Ca^2+^ signals, such as local Ca^2+^ fluctuations or Ca^2+^ waves, to execute their pathological or physiological functions, including the secretion of neurotrophic or neurotoxic factors [102]. The endoplasmic reticulum (ER) is widely regarded as the most crucial and metabolically relevant reservoir and buffering system for intracellular Ca^2+^. It has been recognized that perturbations in astrocyte Ca^2+^ homeostasis can exert toxic effects on ALS motor neurons, but the exact mechanism remains complex and elusive [103]. Recent findings suggest that alterations in astrocyte store-operated Ca^2+^ entry (SOCE) might underlie abnormal gliotransmitter secretion and astrocyte-mediated neurotoxicity in ALS [104,105]. In this study, SOCE in *SOD1^G93A^* mouse spinal cord primary astrocytes was compared with that in wild-type (WT) controls, revealing an increase in SOCE in *SOD1^G93A^* astrocytes concurrent with a decline in ER Ca^2+^-ATPase and ER Ca^2+^ concentrations, resulting in abnormally high intracellular Ca^2+^ variations that potentially harm MNs [105]. Another study [6] found that astrocytes with TDP-43 inclusions exhibit reduced monocarboxylate transporter one and noradrenergic cAMP and Ca^2+^ signaling. These changes play a pivotal role in modulating cellular metabolism, contributing to excessive accumulation of lipid droplets and increased glycolysis and lactate. These findings indicate that astrocytes with TDP-43 inclusions are unable to support neurons. Other astrocyte ion imbalances, such as K^+^ and Na^+^, leading to motor neuron death, are also observed in ALS [106,107,108,109].

### 3.3. PolyP

Polyphosphate (polyP), an inorganic neuroactive compound that potentiates the activity of Nav and Kv channels, is synthesized by astrocytes, functioning as a glial messenger to facilitate communications between astrocytes and neurons [110]. Elevated polyP levels were observed in induced pluripotent stem cell (iPSC)-derived astrocytes from mice and postmortem patients with various ALS/FTD-associated mutations (*SOD1*, *TARDBP*, and *C9ORF72*) [89,90]. Similarly, spinal cord sections from patients with familial and sporadic ALS displayed abundant polyP staining signals [89]. Furthermore, polyP levels were increased in the astrocyte-conditioned media (ACM) from ALS/FTD. In contrast, motor neuron death was significantly decreased by degradation or neutralization of polyP within ALS/FTD astrocytes or ACM, suggesting that excessive astrocytic polyP could be a critical factor for non-cell autonomous MN degeneration and a potential therapeutic target for ALS/FTD [89,90]. Elevated polyP levels were also detected in the cerebrospinal fluid (CSF) of ALS patients, indicating that polyP might serve as a novel biomarker for ALS/FTD [89]. However, it is noteworthy that in an in vivo experiment [89], injecting viral vectors into the intracerebroventricular compartments of *SOD1^G93A^* suckling mice to disrupt polyP production did not delay disease onset or extend the survival of *SOD1^G93A^* mice, despite a significant reduction in polyP deposition in astrocytes and neurons, suggesting that astrocyte-derived polyP might be involved in ALS pathogenesis in conjunction with other factors. Consequently, further exploration and research into the role of polyP in ALS pathogenesis are warranted.

### 3.4. Glutamate

Glutamate (Glu), a major excitatory neurotransmitter in the CNS [111], exhibits overexcitation and excitotoxic effects when its inter-synaptic concentration is abnormally high. This occurs through the activation of glutamate receptors in the postsynaptic membrane, potentially leading to irreversible neuronal damage. Astrocytes play a role in the metabolism of glutamate released into the synaptic cleft [112]. As there is a lack of extracellular glutamate metabolizing enzyme, glutamate can only be taken up by astrocytes with the assistance of glutamate transporters to maintain normal glutamate levels in the extracellular fluid. Five types of excitatory amino acid transporters (EAATs) have been identified, with EAAT1 and EAAT2 primarily found in astrocytes and the other three mainly in neurons. EAAT2, also known as glutamate transporter 1 (GLT-1), accounts for over 90% of glutamate uptake into the cell [113,114]. Under physiological conditions, astrocytes convert glutamate to glutamine, providing energy for motor neurons, serving as a neurotransmitter precursor, and contributing to the amino acid balance in the central nervous system [115]. In many animal models (ALS *SOD1^G93A^* mouse and rat models, as well as TDP43 ALS mouse models) and patients with ALS, a significant reduction in the expression of the *EAAT2* gene in reactive astrocytes has been observed, leading to an accumulation of excess glutamate in the extracellular synaptic cleft. This results in excitatory cytotoxicity of ALS spinal cord motor neurons and impaired motor neuron survival [112,115]. Riluzole, the first drug approved by the FDA for the treatment of ALS, functions by reducing neuronal excitability by blocking glutamatergic neurotransmission in the CNS and activating postsynaptic glutamate receptors to promote glutamate uptake [13].

Previously, membralin (Tmem259 or C19orf6), an innovative component of the ER-associated degradation (ERAD) machinery, was identified, significantly reducing Aβ production by limiting the excessive activation of the γ-secretase complex. Recently, a study has demonstrated a significant reduction in the expression of membralin in astrocytes from the spinal cord of ALS postmortem patients and *SOD1^G93A^* mice [112]. Furthermore, the absence of membralin has been found to significantly impact *EAAT2* expression through the TNF-α/TNFR1/NFκB pathway, dramatically increasing extracellular glutamate and glutamatergic motor neuron toxicity [112]. Conversely, the elevation of membralin expression through transduction of adeno-associated virus (AAV)-membralin in *SOD1^G93A^* mice has demonstrated that increased membralin expression can reverse the neurotoxic effect, prolong mouse survival, reduce glial cell proliferation, and enhance *EAAT2* expression [112]. Collectively, these findings underscored the crucial role of membralin in astrocyte-regulated glutamate homeostasis and EAAT2-mediated glutamate excitotoxicity in ALS.

Previous research has demonstrated that the treatment of astrocytes expressing the *SOD1^G93A^* mutation, when co-cultured with motor neurons in the presence of glutamate, leads to decreased levels of lactate, creatine, creatinine, deoxycarnitine, L-acetylcarnitine, and nicotinamide adenine dinucleotide (NAD) and elevated glucose levels [116]. As NAD is essential for both glycolysis and lactate dehydrogenase activity, the observed reduction in lactate and increased glucose levels in ALS can be attributed to impaired glycolysis resulting from reduced NAD levels [116,117].

### 3.5. Fatty Acids

In the CNS, surplus fatty acids are primarily stored in astrocytes, predominantly as lipid droplets. This storage increases under conditions of hypoxia, cellular stress, and exposure to high levels of exogenous free fatty acids [118]. When released from the ApoE-positive lipid granules of overactive neurons, toxic lipids are taken up by neighboring astrocytes via endocytosis. These transferred fatty acids are utilized as metabolic intermediates to enhance mitochondrial oxidation and detoxification in astrocytes, thereby preventing the accumulation of toxic fatty acids in neurons.

It is well established that the brain is an energy-intensive organ primarily fueled by glucose. However, recent studies have indicated that approximately 20% of the brain’s total energy requirement is derived from fatty acid β-oxidation in astrocytes [118]. Notably, fatty acid oxidation for energy generation is a double-edged sword, as it produces more energy but consumes more oxygen, potentially exposing cells to oxidative stress and exacerbating the production of reactive oxygen species if fatty acid β-oxidation persists [119]. Previous studies have demonstrated that lipids served as the primary energy in ALS due to the high energy demands of neurons and impaired glucose metabolism. However, as mentioned above, this metabolic switch may generate additional oxidative stress products, leading to the demise of motor neurons [120,121]. One study found that astrocytes expressing mutant TDP43 exhibit a more significant accumulation of lipid droplets [6].

Fatty-acid-binding proteins (FABPs) are vital regulators of lipid metabolism, energy homeostasis, and inflammation by controlling nuclear receptor uptake, transport, and ligand availability [122]. FABP7, the predominant brain FABP isoform [123], is primarily expressed in astrocytes, safeguarding these cells against ROS toxicity through the formation of lipid droplets [124] and playing an essential role in reactive astrocyte proliferation associated with CNS injury [125]. Moreover, FABP7 regulates astrocyte responses to external stimuli by controlling lipid raft function [126]. These findings suggest that FABP7 exhibits neuroprotective effects in reactive astrocytes [127]. However, in the spinal cords of *SOD1^G93A^* and *SOD1^H46R/H48Q^* mice, FABP7 expression is upregulated in grey matter astrocytes. It ultimately harms motor neuron survival by promoting NF-κB-driven pro-inflammatory responses in astrocytes [122]. 

In the CNS, polyunsaturated fatty acids, particularly arachidonic acid, are primarily produced and secreted by astrocytes, which further synthesize prostaglandin E2 (PGE2), an inflammatory molecule contributing to neuroinflammation and motor neuron death. The levels of PGE2 in the CSF of most ALS patients are elevated 10-fold [128,129,130,131,132]. Cyclooxygenase 2 (COX2) catalyzes the conversion of arachidonic acid to PGE2; thus, blocking COX2 specifically with celecoxib or rofecoxib may slow down the development and progression of ALS [133,134].

Astrocytes represent the predominant cell type for cholesterol production in the CNS, with their biosynthesis being governed by the transcription factor sterol regulatory element-binding protein-2 (SREBP2), which is notably elevated in ALS [133]. Overexpression of SREBP2 in the CNS results in an accumulation of cholesterol and neutral lipids and the emergence of ALS-like symptoms, including progressive hind limb paralysis, spasticity, and shortened lifespan in mice [120,133].

Recent findings also indicated that astrocyte-mediated cell death is triggered by astrocytes’ secretion of saturated lipids. Furthermore, in vitro and in vivo models of acute axonal injury induced by astrocytes can be mitigated by explicitly silencing the expression of the saturated lipase *ELOVL1* in astrocytes, thereby preventing the formation of long-chain saturated lipids [135]. Increased expression of astrocyte *ELOVL1* or an elevation in the production of long-chain saturated free fatty acids has been reported in ALS, warranting further investigation [133].

## 4. Elements Leading to Astrocyte Activation in ALS

**Figure 1:**
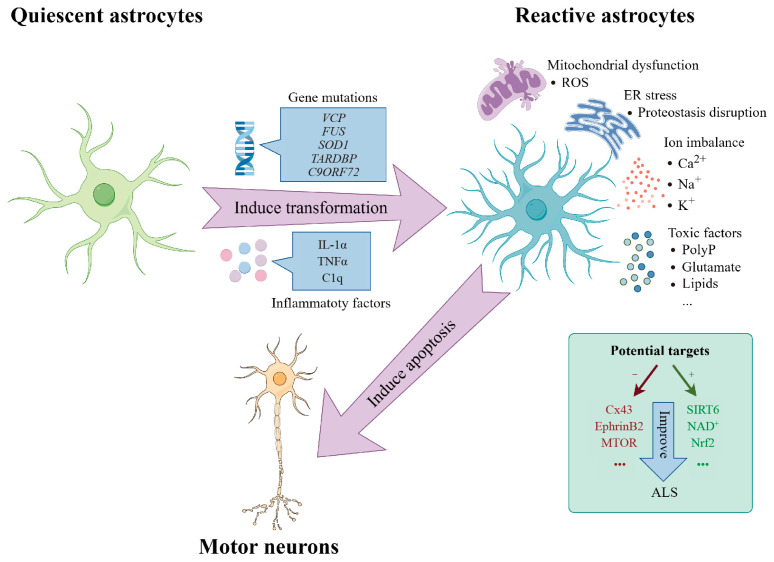
Astrocytes transforming from quiescence to reactivity are toxic to MNs in ALS. Under conditions of gene mutations (*VCP*, *FUS*, *SOD1*, *TARDBP*, *C9ORF72*) or inflammatory factors (IL-1α, TNFα, C1q) in in vivo or in vitro models, astrocytes transform from quiescence to reactivity in ALS. Reactive astrocytes, ultimately resulting in the apoptosis of motor neurons (MNs), are characterized by mitochondrial dysfunction, ER stress, ion imbalance, secretion of toxic factors, etc. Additionally, inhibiting Cx43, EphrinB2, and MTOR or enhancing SIRT6, NAD^+^, and Nrf2 can mitigate motor neuron loss.

In general, the activation of inflammatory factors, upregulation of peroxiredoxin 6 (*PRDX6)*, and gene mutation in astrocytes are the main contributors to the reactive transformation of astrocytes in ALS (Figure 1). This transformation is mediated by pro-inflammatory factors, such as IL-1α, TNFα, and C1q, secreted by reactive microglia. These factors trigger the conversion of quiescent astrocytes into reactive astrocytes, characterized by a notable upregulation of C3 expression, leading to the death of neurons and oligodendrocytes [22]. In the *IL-1α*−/− *TNFα*−/− *C1q*−/− *SOD1^G93A^* mouse model, the knockout of *IL-1α, TNFα*, and *C1q* significantly reduced the proportion of C3^+^ reactive astrocytes, improved motor function, and extended survival in *SOD1^G93A^* mice [83]. The treatment of low doses of IL-1α, TNFα, and C1q to *SOD1^G93A^* -expressing astrocytes and WT astrocytes elicited a notable increase in immune activation and astrocyte reactivity-associated genes upregulated in *SOD1^G93A^* astrocytes [83]. This finding suggests that *SOD1^G93A^* astrocytes can generate a significant response to minor damage. Concurrently, it underscores the crucial role of mutant *SOD1* in transforming astrocytes into reactive astrocytes and demonstrates the cellular autonomy of astrocytes [83]. Furthermore, a recent study has shown that the expression of *PRDX6* in the spinal cord of SOD1^G93A^ mice is also involved in the induction of A1-type astrocytes and the excessive production of inflammatory cytokines through a calcium-dependent phospholipase A-dependent mechanism [84].

Apart from ALS-linked pathogenic variants in *SOD1* [136,137], astrocytes undergo a reactive transformation in response to various pathogenic mutations, such as *VCP*, *FUS*, *TARDBP*, and *C9ORF72* [138,139]. Despite their molecular and functional heterogeneity at early stages, treatment with inflammatory factors ultimately transforms all astrocytes into C3-related reactive astrocytes. In contrast, when WT astrocytes were treated with a conditioned medium obtained from *SOD1* or *VCP* mutant hiPSC-derived astrocytes, no increase in C3 expression or reactive astrocyte transformation was observed [19]. This suggests that the transformation is not caused by the secretion of mutant astrocytes but rather by the autonomy of these cells, further emphasizing the significance of the mutation in the pathogenesis of the disease [19,139]. Moreover, inhibiting the expression of both mutant *SOD1* and wild-type *SOD1* in FALS/SALS astrocytes using a lentivirus encoding a short hairpin (sh) RNA leads to a remarkable reduction in astrocyte-mediated motor neuron toxicity [82,87]. This finding implies that suppressing *SOD1* expression in hiPSC-derived astrocytes could be a potential therapeutic target for both FALS patients with *SOD1* mutations and SALS patients. The newly approved drug tofersen, an antisense oligonucleotide that reduces SOD1 protein synthesis, holds promise [14].

Although the role of reactive astrocytes in the pathogenesis of ALS has been highlighted, it is important to note that they are not a trigger for MN death but crucial contributors. Therefore, the occurrence of neurodegeneration depends on the pathology of neurons.

## 5. Potential Targets on Astrocytes for the Treatment of ALS

At present, the available treatment options and their therapeutic effects for ALS are minimal. As previously mentioned, astrocytes play an essential role in the pathogenesis and disease progression of ALS. To date, riluzole remains the sole drug that targets astrocytes [13]. Therefore, there is an urgent imperative to identify novel potential therapeutic targets for astrocytes.

### 5.1. GDNF

The neurotrophic factor glial-cell-line-derived neurotrophic factor (GDNF), secreted by astrocytes, plays a crucial role in neuronal survival and synaptic promotion. However, the function of reactive astrocytes is impaired in ALS models and patients, resulting in motor neuron death. Consequently, delivering CNS GDNF or transplanting healthy astrocytes may potentially improve motor function in ALS patients [140,141].

A combination of stem cell and gene therapy was employed in a phase I/IIa clinical trial led by Dr Clive Svendsen’s team [140]. Neural progenitor cells, which were genetically engineered to express GDNF protein, were transplanted into the dorsal and ventral horns of the lumbar segment of the spinal cord in ALS patients. These cells were then transformed into supportive glial cells. The neural precursor cells can give rise to new supporting glial cells, releasing the protective protein GDNF, collectively aiding in preserving motor neurons [140]. This “double whammy” approach concurrently employs the generated new glial cells and GDNF protein to support the survival of dying motor neurons in the face of the disease.

The limited half-life of GDNF in plasma, its inability to directly cross the blood–brain barrier during subcutaneous administration, and its poor penetration into the brain and spinal cord during intrathecal injection trials render it challenging to achieve a therapeutic effect using these approaches. Consequently, in this trial [140], the stem cell product CNS10-NPC-GDNF was safely delivered into the dorsal and ventral horns of the lumbar segment of the spinal cord in ALS patients using a novel in-house-developed injection device. After a single transplant via this innovative method, neural progenitor cells survived up to 42 months and continued to generate new glial cells and GDNF proteins. The results indicated that the rate of leg strength decline was slower on the treated side than on the untreated side, although this difference was not statistically significant. Furthermore, this cell transplantation did not cause substantial adverse effects on muscle strength in the treated leg compared to the untreated side. However, in some patients, many of these cells reached sensory areas in the spinal cord, potentially leading to pain [140]. Overall, this clinical trial demonstrated the safety of this approach, but further assessments of efficacy are required. The team is also currently utilizing these GDNF-secreting stem cells in another ALS clinical trial (https://clinicaltrials.gov/ct2/show/NCT05306457, accessed on 3 November 2023) by transplanting them into the “hand-knob” area of the motor cortex of patients with ALS. Ongoing progress in the efficacy and safety of stem cell combined gene therapy for ALS patients should be expected.

### 5.2. AstroRx^®^

AstroRx^®^, an allogeneic cell-based product derived from human embryonic stem cells, is generated under cGMP conditions in Kadimastem’s GMP facility via standard procedures and assessed according to stringent criteria by external qualified certified GLP laboratory (Hylabs laboratories, Jerusalem, Israel) [141]. It exhibits functional, healthy astrocyte effects, such as clearing excessive glutamate, reducing oxidative stress, secreting various neuroprotective factors, and acting as an immunomodulator. In a phase I/IIa clinical trial involving intrathecally injected human astrocytes (AstroRx^®^), the rate of ALSFRS-R worsening within the first three months post-treatment was significantly reduced, accompanied by fewer adverse events, regardless of whether subjects received high or low doses of healthy astrocytes. These positive results warrant further exploration of repeated intrathecal administration of AstroRx^®^, such as every three months [141].

Regarding the two emerging clinical trials mentioned above, each has its merits and drawbacks. After a single treatment with stem cells combined with gene therapy, neural progenitor cells can survive for extended periods, differentiate into new glial cells, continuously produce GDNF proteins, and directly act on a specific group of neurons. In contrast, direct intrathecal injection of astrocytes has a shorter duration of action and may necessitate injections every three months. Most importantly, the ability of healthy astrocytes to perform their normal function in the CNS within an inflammatory environment remains unclear. Meanwhile, there are existing challenges associated with stem cell transplantation, including immune rejection, abnormal hyperplasia, ethical concerns, etc. In terms of efficacy, intrathecal injection of astrocytes has been preliminarily validated in phase I/IIa clinical trials. However, the effect of stem cells combined with gene therapy remains uncertain in current clinical trials. Overall, these research advancements are promising and deserve further investigation.

### 5.3. Cx43

In recent years, foundational research has unearthed additional potential astrocyte targets. Cx43, an essential astrocyte connectivity protein, together with its hemichannels, facilitates communication between astrocytes within the central nervous system [142]. Increased expression of Cx43 has been observed in animal models of ALS, the cerebrospinal fluid of ALS patients, and postmortem samples, indicating its toxicity towards neurons [143]. In vitro experiments such as co-culturing and blocking Cx43 and its hemichannels corroborate this. In vivo experiments have revealed that the removal of Cx43, specifically from astrocytes in *SOD1^G93A^* mice, resulted in a spatial (in the cervical and lumbar spinal cords) and temporal (at the pre-symptomatic, symptomatic, and end stages) deceleration of disease progression, as well as protection for motor neurons, and an increase in survival rate [143]. Tonabersat, a drug candidate capable of blocking Cx43 hemichannels and crossing the blood–brain barrier [144], has been shown to provide neuroprotection by reducing neuronal death when co-cultured with human induced pluripotent stem-cell-derived astrocytes (hiPSC-A) derived from both familial and sporadic ALS patients using control motor neurons (hiPSC-MNs) [144]. Administration of tonabersat intraperitoneally at 10 mg/kg once daily to *SOD1^G93A^* mice exhibited potential for enhancing motor function [143]. Notably, the expression of Cx43 in astrocytes remains unaltered by tonabersat, whereas the expression of GFAP and Iba-1 significantly decreased [143]. The drug has also been investigated in the context of migraine and epilepsy [144]. In conclusion, the targeted blockade of astrocyte Cx43 and the integration of tonabersat into ALS clinical trials are worth considering.

### 5.4. EphrinB2

Expression of ephrinB2, a transmembrane signaling molecule, is significantly elevated in astrocytes within the spinal cord of *SOD1^G93A^* mice and ALS patients [145,146]. Delivery of viral-mediated shRNA to astrocytes in the cervical segment of the spinal cord selectively represses ephrinB2 expression, thereby mitigating motor neuron loss and preserving respiratory function by sustaining motor neuron innervation of the diaphragm [146]. This study suggests that the upregulation of ephrinB2 is both a transcellular signaling mechanism for astrocyte pathogenicity in ALS and a promising therapeutic target.

### 5.5. NAD^+^, Nrf2, and SIRT6

Preliminary findings suggest that NAD^+^, Nrf2, and SIRT6 synthesized by astrocytes confer neuroprotection against ALS, with SIRT6 playing a pivotal role. An increase in NAD^+^ availability is known to enhance resistance to oxidative stress and reduce mitochondrial ROS production in various cell types and disease models [147]. Nrf2 activation is critical for regulating antioxidant defenses and protecting neighboring neurons in co-culture and in vivo settings [148,149]. Furthermore, elevating total NAD^+^ levels in astrocytes activates Nrf2 and SIRT6 in these cells, while SIRT6 overexpression further activates Nrf2. Decreased expression of NAD^+^, Nrf2, and SIRT6 has been observed in the spinal cords of ALS patients. In animal models of ALS, NAD^+^ depletion does not affect survival, but administering biologically active NAD^+^ precursors significantly improves motor performance and extends survival [150]. In addition, upregulating Nrf2 in astrocytes has been demonstrated to promote neuronal survival in in vitro co-culture studies [151] and in an ALS mouse model [152]. However, silencing SIRT6 expression in an in vitro cell culture model did not prevent astrocyte neurotoxicity towards motor neurons, even with pre-supplementation of NAD^+^ precursors [153]. Thus, SIRT6 plays a crucial role in this neuroprotective effect. Overall, enhancing SIRT6 and Nrf2 activity and administering NAD^+^ precursors that abolish the neurotoxic phenotype of astrocytes expressing the ALS-associated mutation *SOD1* are potential therapy approaches [154,155,156].

### 5.6. MTOR

The mechanistic target of rapamycin kinase (MTOR) is a regulator of numerous extracellular and intracellular signals that participate in cellular metabolism, growth, proliferation, survival, and macro-autophagy/autophagy [157]. Activation of the MTOR pathway has been demonstrated to be elevated in *SOD1^G93A^* mutant hiPSC-derived astrocytes, resulting in the suppression of macro-autophagy/autophagy, aberrant cell proliferation, and an increased reactivity of the astrocytes [158]. Concurrently, MTOR pathway activation is correlated with post-transcriptional upregulation of the insulin-like growth factor 1 receptor (IGF1R). Therefore, inhibition of the IGF1R-MTOR pathway decreases cell proliferation and the reactivity of mutant *SOD1^G93A^* astrocytes, thereby mitigating their toxicity towards motor neurons. These findings suggest that modulation of the IGF1R-MTOR pathway in astrocytes may represent a plausible therapeutic target for ALS [158].

## 6. Conclusions and Future Prospects

The pathogenesis of ALS is complex and involves multiple pathophysiological mechanisms, among which reactive astrocytes play a crucial role. Therefore, therapeutic strategies targeting astrocytes, such as inhibiting their reactive transformation at each stage of ALS, obstructing the pathways through which transformed reactive astrocytes exert toxic effects on neurons, and replacing reactive astrocytes with normal astrocytes, could potentially offer ALS patients the prospect of prolonged survival and improved motor and respiratory functions.

Despite extensive research confirming the non-cell-autonomous functions of reactive astrocytes in ALS, numerous queries necessitate future investigation. These encompass: (1) the practical applications of these findings for ALS patients; (2) the development of more precise and scientifically grounded classification methods to identify reactive astrocytes in ALS; (3) investigation into potential variations in morphology, molecular composition, functionality, and gene expression of reactive astrocytes among different clinical subtypes of ALS. Understanding these distinctions could offer insights into disease onset, affected regions, progression, motor function impairment, respiratory function decline, prognosis, and other aspects. (4) By utilizing advanced techniques such as positron emission tomography (PET), specific markers and tracers with defined functions can be employed to dynamically monitor the changes and migration patterns of astrocytes within various regions of the central nervous system during pre-symptomatic stages as well as symptomatic and end-stage phases in both animal and human patients with ALS. This approach aims to visualize the involvement of reactive astrocytes directly in living organisms throughout the progression of ALS. It is anticipated that further comprehensive research will address these inquiries while enhancing our understanding of the role of reactive astrocytes in ALS.

## Figures and Tables

**Table 1:** Comparison between healthy astrocytes and reactive astrocytes.

Characteristic	Healthy Astrocytes	Reactive Astrocytes
Morphology	Star-shaped morphology	Hypertrophy
	Multiple branches with numerous fine processes	Process elongation
		Overlap of some structures in three-dimensional space
Molecular Aspect	Low GFAP expression	Increased GFAP expression
Biochemistry	Release of neurotrophic factors	Increased release of pro-inflammatory factors
		Increased production of ROS
		Activated complement cascades
Transcriptional Regulation	Steady-state regulation of gene expression	Upregulation of genes associated with neuroinflammation
Function	Neuron trophic support	No or decreased trophic support or active neurotoxicity
	Neurotransmitter uptake and recycling	Decreased neurotransmitter uptake and/or recycling
	Synapse formation, maturation, and function	Decreased synapse formation and altered neuronal activity
	Regulation of blood and glymphatic flow	Increased immune cell infiltration and blood–brain barrier maintenance and/or repair
	Interaction and coordination with immune cells	Proliferate and form scars or borders
	Stable and rhythmic calcium transients	Corral peripheral immune cells and/or amplify inflammatory responses
		Irregular calcium transients, decreased gap junction coupling
		Abnormal cellular metabolism
		Newly acquired neurotoxic or neuroprotective functions, depending on context

**Table 2:** Comparison between A1 astrocytes and A2 astrocytes.

Characteristic	A1 Astrocytes	A2 Astrocytes
Morphology	Hypertrophy, long dendrites	Hypertrophy, few dendrites
Marker	C3, GBP2, Serping1	PTX3, S100a10, SphK1, tm4sf1, S1Pr3, Tweak
Signaling pathway	Activated NF-κB, JAK/STAT3	Activated PK2/PKR1, JAK/STAT3, FGF2/FGFR1, CXCR7/PI3K/Akt
Cellular functions	Neurotoxic effect: upregulate pro-inflammatory factors; associated with neurodegeneration and chronic neuropathic pain	Neuroprotective effect: upregulate neurotrophic factors and pro-synaptic thrombospondins; promote neuronal growth and support synaptic repair

## Data Availability

Not applicable.

## Abbreviations

BBB: blood–brain barrier; ALS: amyotrophic lateral sclerosis; SALS: sporadic amyotrophic lateral sclerosis; FALS: familial amyotrophic lateral sclerosis; FTD: frontotemporal dementia; CNS: central nervous system; GFAP: glial fibrillary acidic protein; MNs: motor neurons; AD: Alzheimer’s disease; MS: multiple sclerosis; PD: Parkinson’s disease; LPS: lipopolysaccharide; TRAIL: TNF-related apoptosis-inducing ligand; NDs: neurodegenerative diseases; HD: Huntington’s disease; ROS: reactive oxygen species; SOD1: superoxide dismutase-1; ER: endoplasmic reticulum; SOCE: store-operated Ca^2+^ entry; polyP: polyphosphate; ACM: astrocyte conditioned media; iPSC: induced pluripotent stem cell; FTD: frontotemporal dementia; TARDBP: transactive response DNA-binding protein; C9ORF72: chromosome 9 open reading frame 72; CSF: cerebrospinal fluid; EAATs: excitatory amino acid transporters; GLT-1: glutamate transporter 1; ERAD: ER-associated degradation; NAD: nicotinamide adenine dinucleotide; FABPs: fatty-acid-binding proteins; PGE2: prostaglandin E2; COX2: cyclooxygenase 2; SREBP2: sterol regulatory element-binding protein-2; PRDX6: peroxiredoxin 6; MTOR: mechanistic target of rapamycin kinase; IGF1R: insulin-like growth factor 1 receptor; SIRT6: sirtuin 6; Nrf2: nuclear factor erythroid 2-related factor 2; NPCs: neural progenitor cells; GDNF: glial-cell-derived neurotrophic factor; Cx43: connexin 43; VCP: valosin-containing protein; TDP-43: TAR DNA binding protein 43; FUS: fused in sarcoma; iPSC: induced pluripotent stem cell; LPS: lipopolysaccharide; C3: complement component 3; TNFSF 10: tumor necrosis factor superfamily member 10; EAE: experimental autoimmune encephalomyelitis; PbAc: lead acetate; PARK7/DJ-1: Parkinson’s disease protein 7; MC: motor cortex; SC: spinal cortex; DCA: dichloroacetate; PDH: pyruvate dehydrogenase; WT: wild type; Glu: glutamate.
